# Co‐infection with *Nocardia* spp. in a patient wit*h Mycobacterium avium* complex pulmonary disease: A case report

**DOI:** 10.1002/rcr2.1036

**Published:** 2022-09-07

**Authors:** Nozomi Tokita, Naohisa Urabe, Susumu Sakamoto, Asuka Yamaguchi, Ryo Sekiguchi, Kazuma Kishi

**Affiliations:** ^1^ Department of Respiratory Medicine Toho University Omori Medical Center Tokyo Japan

**Keywords:** co‐infection, *Mycobacterium avium* complex, Nocardia species, nontuberculous mycobacterium

## Abstract

*Mycobacterium avium complex* pulmonary disease (MAC‐PD) is sometimes accompanied by co‐infection with other pathogenic microorganisms such as *Pseudomonas aeruginosa* and *Haemophilus influenzae*. However, co‐infection with *Nocardia* spp. has been rarely reported. We report on a patient diagnosed as having co‐infection with *Nocardia* after treatment for MAC‐PD, which was successfully treated using trimethoprim‐sulfamethoxazole (TMP‐SMX). A 74‐year‐old woman with MAC‐PD was admitted to our hospital to undergo re‐examination for pathogenic microorganisms because chest computed tomography (CT) findings did not improve after treatment for MAC‐PD. She underwent bronchoscopy and *Nocardia spp.* was detected from bronchoalveolar lavage fluid culture. Chest CT findings improved after 6 months of treatment using TMP‐SMX. Co‐infection with other pathogenic microorganisms should be considered when chest CT findings worsen after adequate treatment of MAC‐PD. Chest CT findings of *Nocardia* pulmonary disease in immunocompetent patients can mimic those of MAC‐PD and should therefore be differentiated one from the other.

## Graphical abstract

We report on a case of Mycobacterium avium complex pulmonary disease (MAC‐PD) with Nocardia spp. co‐infection after treatment for MAC, where chest computed tomography (CT) findings showed improvement after treatment with trimethoprim‐sulfamethoxazole (TMP‐SMX).

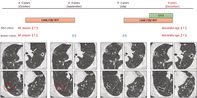

## INTRODUCTION

Patients with *Mycobacterium avium* complex pulmonary disease (MAC‐PD) sometimes develop co‐infection with other pathogenic microorganisms.^1^ However, co‐infection with *Nocardia* spp. has rarely been reported. *Nocardia* spp. are naturally‐occurring normal and environmental commensal bacteria found ubiquitously in water and soil worldwide. *Nocardia* pulmonary disease (*Nocardia*‐PD) is a rare infectious disease affecting immunocompromised patients but is also known to occur in immunocompetent patients. We report on a case of MAC‐PD with *Nocardia* spp. co‐infection after treatment for MAC, where chest computed tomography (CT) findings showed improvement after treatment with trimethoprim‐sulfamethoxazole (TMP‐SMX).

## CASE REPORT

**FIGURE 1:**
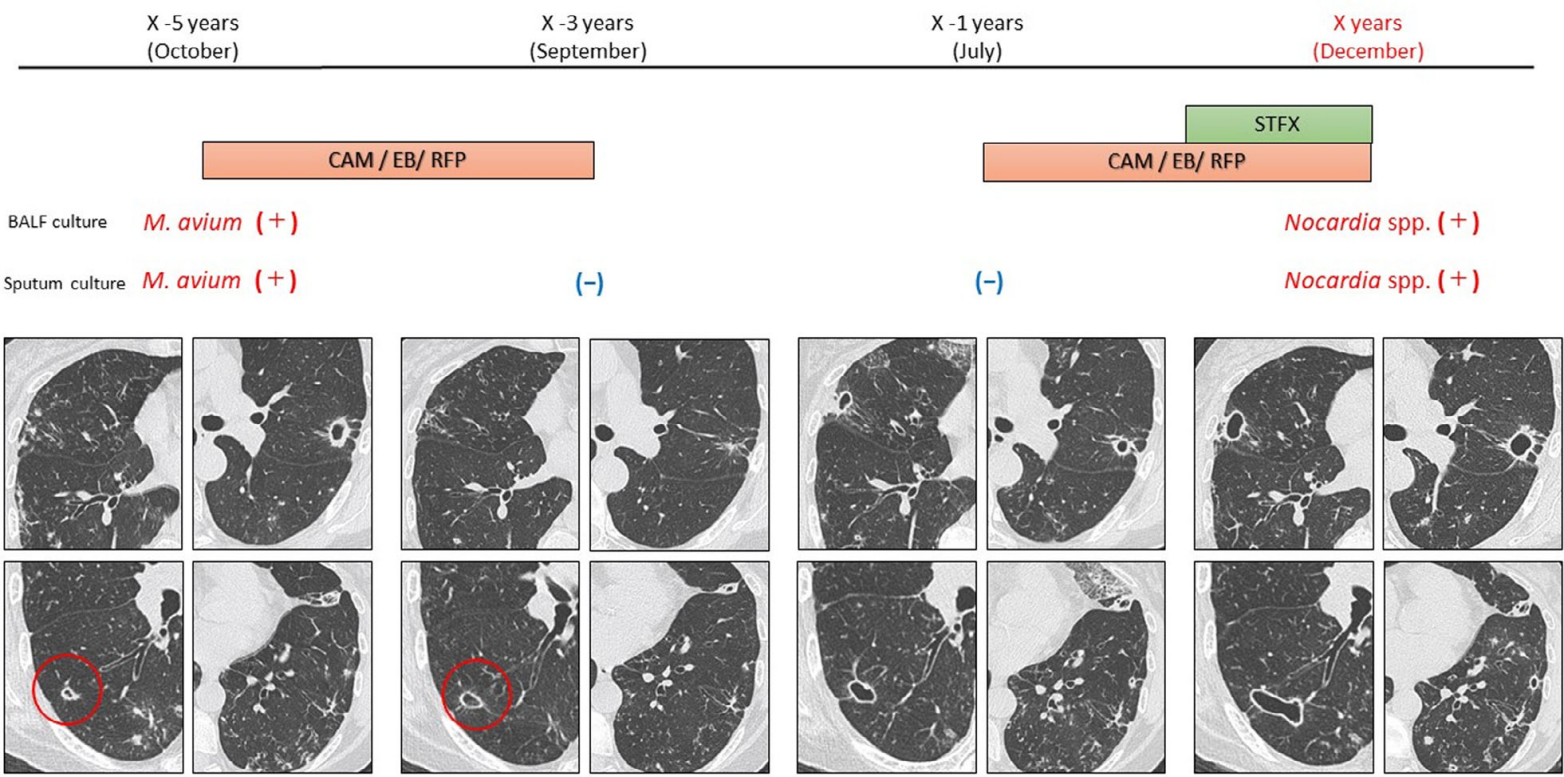
Clinical course of the patient during initial and second treatment for *Mycobacterium avium* complex pulmonary disease. Red circle demonstrates a cavitary lesion in the right lower lobe with no response to treatment. CAM, clarithromycin; EB, ethambutol; RFP, rifampicin; STFX, sitafloxacin

A 74‐year‐old woman with MAC‐PD was admitted to our hospital to undergo re‐examination for pathogenic microorganisms. Five years before this admission, she had been diagnosed with MAC‐PD based on a positive *M. avium* culture from bronchoalveolar lavage fluid (BALF). Chest CT showed bilateral bronchiectasis and cavitary lesions then. Treatment for MAC‐PD was started with clarithromycin (CAM), rifampicin (RFP), and ethambutol (EB) for 2 years. Chest CT showed considerable improvement in most centrilobular nodules and cavities, but one right lower lobe cavitary lesion showed no treatment response (Figure 1). *M. avium* was not detected from sputum culture post‐treatment. She remained stable during follow‐up with no post‐treatment exacerbation for 2 years.

Notably, 17 months before this admission (July, X‐1 years), she developed hemoptysis, and chest radiography had worsened. Chest CT findings revealed increased number of centrilobular nodules and cavitary lesion enlargement (Figure 1). Sputum culture was negative. The same treatment regimen for MAC‐PD was resumed. Although Sitafloxacin (STFX) was added to the treatment regimen at 3 months before this admission, chest CT findings worsened further. Thus, bronchoscopy was planned to re‐evaluate for pathogenic microorganisms.

**FIGURE 2:**
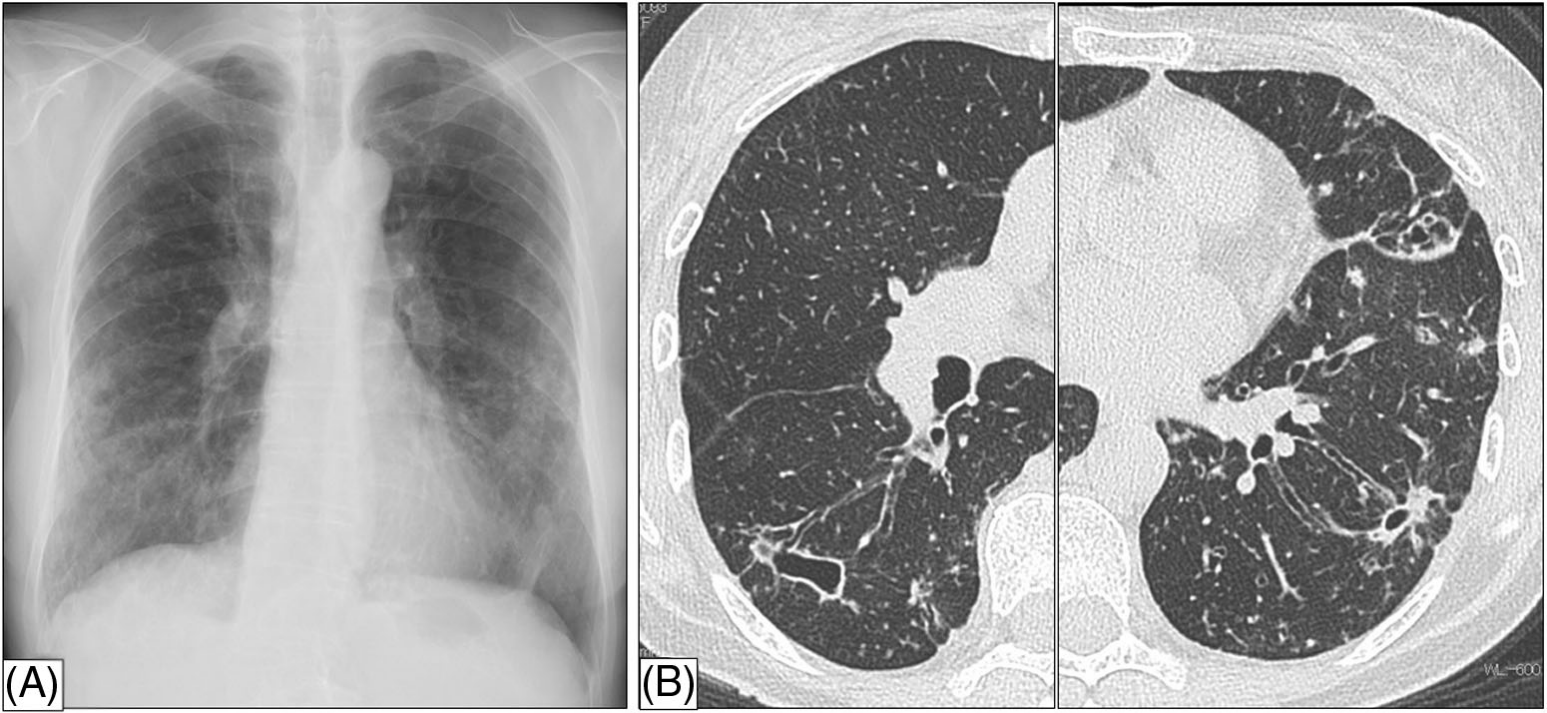
(A) Chest radiograph showing predominantly peripheral opacities and small nodules, bilaterally. (B) High‐resolution CT showing bilateral bronchiectasis, centrilobular nodules, and cavities

She had developed community‐acquired pneumonia at age 54 years, but had no history of dust exposure or allergies; she was a former smoker (7 pack‐years). On admission, temperature was 36.6°C, pulse rate, blood pressure, and respiratory rate were normal; oxygen saturation was 97% in room air. Auscultation revealed a clear chest with normal vesicular breath sounds. Laboratory investigation revealed C‐reactive protein level of 6.0 mg/dL (normal, <0.3 mg/dL), leucocyte count of 12,000cells/μL, and serum β‐D glucan level of <6.0 pg/mL (normal, <10 pg/mL). Serum aspergillus antigen and antibody were negative. HIV serology and interferon‐gamma release assay were negative. Serum anti‐glycopeptidolipid‐core IgA titre was <0.5 U/mL (normal, <0.7 U/mL). Chest radiography showed predominantly peripheral opacities and small nodules bilaterally (Figure 2A). High‐resolution CT showed bilateral bronchiectasis, centrilobular nodules, and cavities (Figure 2B).

**FIGURE 3:**
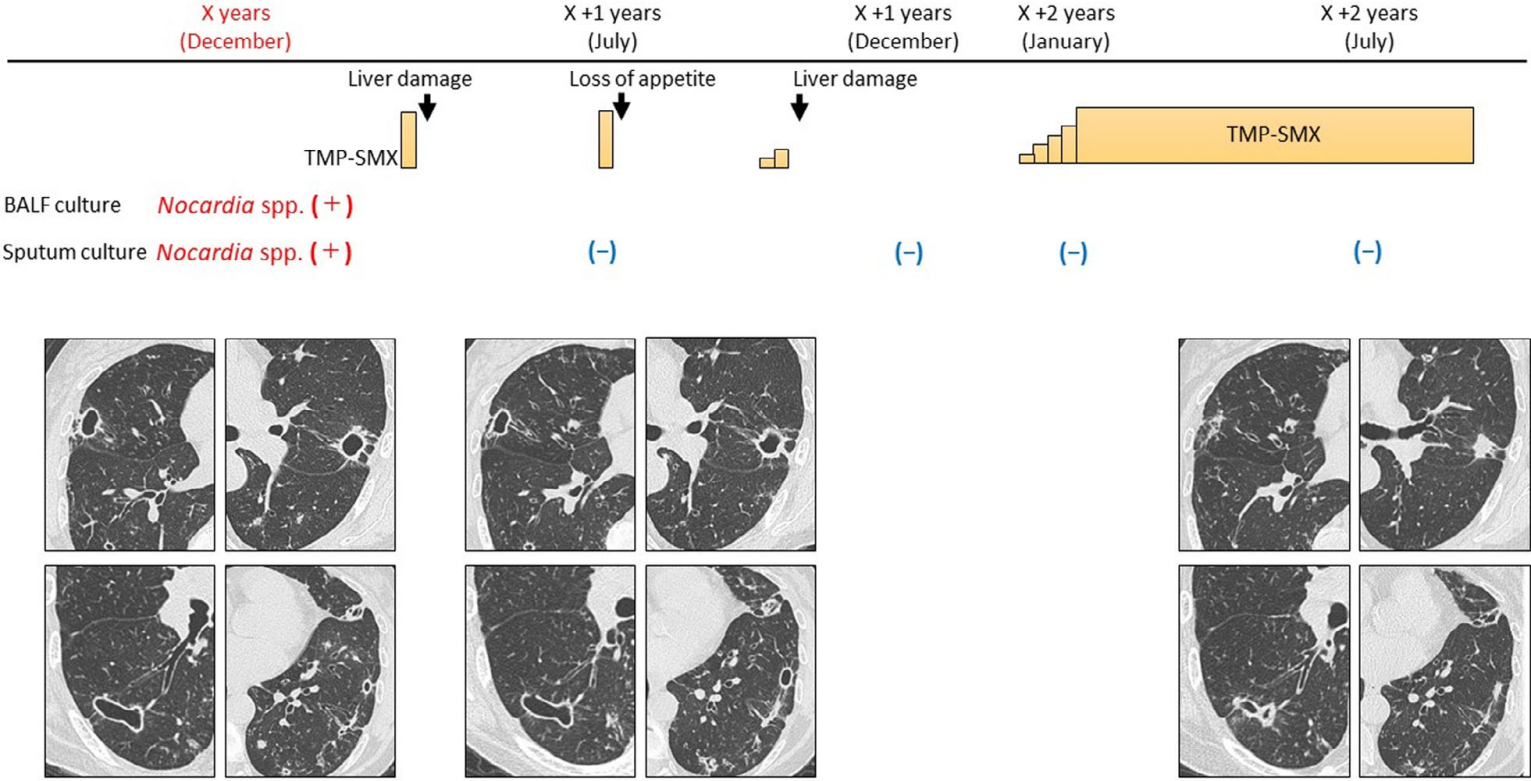
Clinical course of the patient during treatment for *Nocardia* pulmonary disease. TMP‐SMX, trimethoprim‐ sulfamethoxazole; BALF, Bronchoalveolar lavage fluid

*Nocardia* spp. was detected in BALF culture and sputum examination after bronchoscopy. We identified the *Nocardia* spp. by using biochemical methods but could not identify the subspecies. Minimum inhibitory concentration for TMP‐SMX was not examined. Treatment with TMP‐SMX for *Nocardia* was started but was discontinued thrice due to side effects of liver damage or anorexia. About 2 years later, treatment was reinstituted with desensitization to TMP‐SMX. Chest CT findings improved after 6 months of treatment with TMP‐SMX (Figure 3). *Nocardia* spp. was not detected in sputum examination thereafter.

## DISCUSSION

This case highlights two major points. First, co‐infection with other pathogenic microorganisms should be considered when chest CT findings worsen during treatment of MAC‐PD. Second, chest CT findings of *Nocardia*‐PD in an immunocompetent patient can mimic those of MAC‐PD; the two conditions should therefore be differentiated from one another.

Successful treatment of MAC‐PD is typically defined as “sputum culture conversion”, but some patients show worsening chest CT findings even after conversion. There is no time limit for the definitive diagnosis of MAC‐PD, thus when imaging findings are consistent with MAC‐PD and MAC is detected in BALF at least once previously, treatment for MAC may be continued despite co‐infection or bacteria replacement. Also, co‐infection with other pathogenic microorganisms, like *Pseudomonas aeruginosa* and *Haemophilus influenzae*, has been reported in patients with MAC‐PD.^1^ In this case, one cavitary lesion in the right lower lobe did not respond to initial MAC‐PD treatment with subsequent worsening of the entire lung lesions, which improved with treatment for *Nocardia*‐PD suggesting that co‐infection had likely occurred during the initial MAC‐PD treatment. Co‐infection with other pathogenic microorganisms should be considered when chest CT findings worsen during treatment of MAC‐PD.

Chest CT findings of *Nocardia*‐PD in immunocompromised patients frequently include lobar or multifocal consolidation, whereas those in immunocompetent patients can mimic MAC‐PD. A report of pulmonary *Nocardia transvalensis* infection in an immunocompetent patient described chest CT findings showing centrilobular micronodular densities associated with bronchial wall thickening and bronchiectasis.^2^ Another study on *Nocardia*‐PD in 12 immunocompetent patients reported a high incidence of bronchiectasis (67%) and centrilobular nodules (67%) on chest CT; the pattern of these findings was like that of MAC‐PD.^3^ *Nocardia* spp. and MAC share several characteristics. They are ubiquitous, endemic, acid‐fast, and induce granuloma formation pathologically. These similarities may contribute to the similar imaging findings. Some cases of co‐infection with MAC‐PD and *Nocardia* spp. have been reported among immunocompetent patients. Comparing our patient with three previous patients for whom detailed information was available, chest CT findings were bronchiectasis and centrilobular nodular lesions indistinguishable from MAC in all patients.^4^, ^5^ Identification of *Nocardia* spp. was *cyriacigeorgica* in two patients and *farcinica* in one patient. *Nocardia* was identified in the three patients, at the same time as MAC, during untreated MAC follow‐up, and after treatment for MAC as in this case. Two out of three patients were successfully treated with TMP‐SMX.

The primary limitation of this case is the lack of species identification for *Nocardia* spp. The characteristics of *Nocardia* spp. vary among species, and this could affect the clinical course.

In patients with previously diagnosed MAC‐PD for whom treatment for MAC is ineffective, bronchoscopy is recommended to allow reexamination for the causative microorganisms.

## AUTHOR CONTRIBUTIONS

Naohisa Urabe designed the study; Nozomi Tokita drafted the manuscript; Susumu Sakamoto and Asuka Yamaguchi participated in editing the manuscript; Ryo Sekiguchi revised it critically for important intellectual content; Kazama Kishi final approval of the version to be published. All authors read and critically revised the first as well as the subsequent and final drafts of this manuscript. All authors read and approved the final manuscript.

## CONFLICT OF INTEREST

None declared.

## ETHICS STATEMENT

The authors declare that appropriate written informed consent was obtained for the publication of this manuscript and accompanying images.

## Data Availability

The data that support the findings of this study are available from the corresponding author upon reasonable request.
